# Evaluation of the ForenSeq® Kintelligence Kit and the FORensic Capture Enrichment Panel for Unidentified and Missing Persons Casework

**DOI:** 10.1007/s00414-025-03492-4

**Published:** 2025-04-07

**Authors:** Jessica L. Watson, Kelly Grisedale, Dennis McNevin, Jodie Ward

**Affiliations:** 1 Forensics Command, Australian Federal Police, Canberra, Australia; 2 National DNA Program for Unidentified and Missing Persons, Australian Federal Police, Canberra, Australia; 3 Centre for Forensic Science, School of Mathematical & Physical Science, Faculty of Science, University of Technology Sydney, Sydney, Australia

**Keywords:** Targeted amplicon sequencing, Single nucleotide polymorphism, Unidentified human remains, Missing persons, Whole genome sequencing, Kinship

## Abstract

Targeted amplicon sequencing (TAS) employs massively parallel sequencing technology to generate profiles comprised of several thousand single nucleotide polymorphisms (SNPs) to assist in identifying an individual and generating investigative leads. By targeting a range of SNP classes, profiles are able to be analysed to infer biological sex, externally visible characteristics, biogeographical ancestry, paternal lineage and relationships to other individuals. Such leads can be beneficial for human remains identification where antemortem data is not available for comparison. This study evaluated the performance and requirements of two in-house TAS pipelines: the ForenSeq® Kintelligence Kit and the FORensic Capture Enrichment (FORCE) panel. Both TAS pipelines demonstrated suitability for a range of samples typically encountered in missing persons cases, including buccal, bone, tooth and nail samples. There was a high degree of concordance between the TAS genotypes and the majority of the genetic intelligence produced was consistent with the self-declared information provided by DNA donors. This study highlights the requirements for each pipeline to be considered by forensic laboratories seeking to establish a forensic genomics capability for unidentified and missing persons casework.

## Introduction

DNA analysis is one of the primary identification methods employed in unidentified human remains (UHR) investigations due to its ability to differentiate individuals [1–3]. Routine short tandem repeat (STR) profiles are uploaded to a law enforcement DNA database to be compared against known profiles in an attempt to obtain a direct or kinship match [4]. UHR DNA analysis can be complicated by the postmortem interval of the remains, degradation of the DNA and the unavailability of suitable direct or familial reference samples. In addition, STR profiles are only suitable for 1 st or 2nd degree kinship inferences. When routine STR testing and law enforcement database searches do not result in an identification, forensic genomics techniques can be employed to provide new investigative leads. [3]

Single nucleotide polymorphisms (SNPs) are single points of variation in the genome and can be categorised into SNP classes based on the information that can be yielded, including: identity-informative SNPs (iiSNPs) for individualisation; phenotype-informative SNPs (piSNPs) for estimating externally visible characteristics (EVCs); ancestry-informative SNPs (aiSNPs) for estimating biogeographical ancestry (BGA); kinship-informative SNPs (kiSNPs) for detecting close and distant genetic relatives; and sex-chromosome SNPs from the Y chromosome (Y SNPs) and X chromosome (X SNPs) for inferring biological sex and paternal lineage. [5–7]

DNA sequencing techniques have been continuously developed since their first application for forensic identification purposes in order to produce more sensitive, accurate, discriminatory and informative DNA profiles [8–10]. Targeted amplicon sequencing (TAS) employs massively parallel sequencing (MPS) technology, widely used for SNP typing due to its multiplexing power and ability to sequence millions of reads from multiple samples simultaneously [11]. Due to the use of benchtop MPS instruments, TAS is the most easily integrated genotyping technology for an ISO/IEC 17025 accredited forensic laboratory. Despite having lower density SNP coverage than other genomics technologies such as microarray and whole genome sequencing (WGS), forensic TAS panels can target several thousands of SNPs that have been specifically curated for forensic applications. They produce medium density SNP genotypes, as opposed to the high density genotypes produced by microarrays and WGS. Consequently, some privacy risks can be mitigated if medically informative SNPs, sequenced using other approaches, are deliberately excluded. [3, 12]

The ForenSeq® Kintelligence Kit (QIAGEN, Hilden, Germany) and the FORensic Capture Enrichment (FORCE) panel are two TAS kits available for forensic genomics [13–15]. Verogen, Inc. (now a QIAGEN company) released the Kintelligence Kit in 2021 for use on the MiSeq FGx® Sequencing System [16]. This panel was designed for processing a variety of forensic casework samples and targets over ten thousand SNPs. The FORCE panel was originally developed by Tillmar et al*.* (2021) as a hybridisation capture assay for highly degraded contemporary and historical UHR and targets over five thousand SNPs [15]. The FORCE workflow has been subsequently redesigned to be agnostic for multiple library preparation and sequencing chemistries, including Illumina (QIAseq) and Ion Torrent [17, 18] Table 1 compares the number of SNPs by class for the Kintelligence Kit and FORCE panel.

**Table 1:** Number of SNPs by class in the ForenSeq® Kintelligence Kit and the FORCE panel with the QIAseq workflow as well as overlapping SNPs between the panels. [14, 17]

SNP Class	ForenSeq® Kintelligence Kit	FORCE Panel (QIAseq Workflow)	Overlapping SNPs
X SNPs	106	246	2
Y SNPs	85	883	11
piSNPs	24^a^	41^b^	22^d^
aiSNPs	54^a^	254^b^	54^d^
iiSNPs	94	137^c^	93
kiSNPs	9,867	3,936^c^	810
**Total**	**10,230**	**5,497**	**992**

^a^ Two SNPs overlap the piSNP and aiSNP classes in the Kintelligence Kit and are only counted in the piSNPs

^b^ Three SNPs overlap the piSNP and aiSNP classes in the FORCE panel and are only counted in the piSNPs

^c^ Two SNPs overlap in the iiSNP and kiSNP classes in the FORCE panel and are only counted in the kiSNPs

^d^ The two SNPs that overlap the piSNP and aiSNP classes are only counted in the piSNPs

The SNP genotypes produced by the TAS pipelines can be used to assist in identifying an individual and inferring biological sex, EVCs, BGA, paternal lineage and extended kinship. Extended kinship can be conducted in two ways:

1. by examining the number and length of DNA segments shared by two individuals where the segments consist of multi-SNP haplotypes that are identical by descent (IBD), or
2. by calculating a likelihood ratio (LR), which compares likelihoods for two alternative kinship scenario propositions for two individuals based on SNPs that are identical by state (IBS) and their allele frequencies within the population in question. [19–23]

Since 2018, extended kinship analysis has been applied to forensic investigative genetic genealogy (FIGG) to detect close and distant genetic relatives on law enforcement accessible public genetic genealogy databases [24, 25]. A SNP profile of an unknown person can be compared to profiles uploaded by consenting members of the public. FIGG can be a powerful intelligence tool where a person has not been reported as missing, or in cases where antemortem data or family reference samples are not available.

A laboratory should endeavour to validate and accredit a forensic genomics workflow that is best suited for their typical sample types, laboratory capacity, available expertise and intended genetic intelligence applications to advance unresolved casework. This study evaluated in-house TAS pipelines to inform an optimal forensic genomics strategy for the Australian Federal Police (AFP) National DNA Program for Unidentified and Missing Persons. Several reference- and casework-type samples were sequenced using the Kintelligence Kit and the FORCE panel with the QIAseq workflow. These findings may assist other forensic laboratories seeking to establish a SNP typing capability.

## Methods

### Ethics Approval and Sample Preparation

Ethics approval for this forensic genomics research and collection of samples from the Australian Facility for Taphonomic Experimental Research (AFTER) was granted by the University of Technology Sydney (UTS) Human Research Ethics Committee (HREC); UTS HREC NO. ETH21 - 5821 and UTS HREC NO. ETH18 - 2999, respectively.

**Fig. 1:**
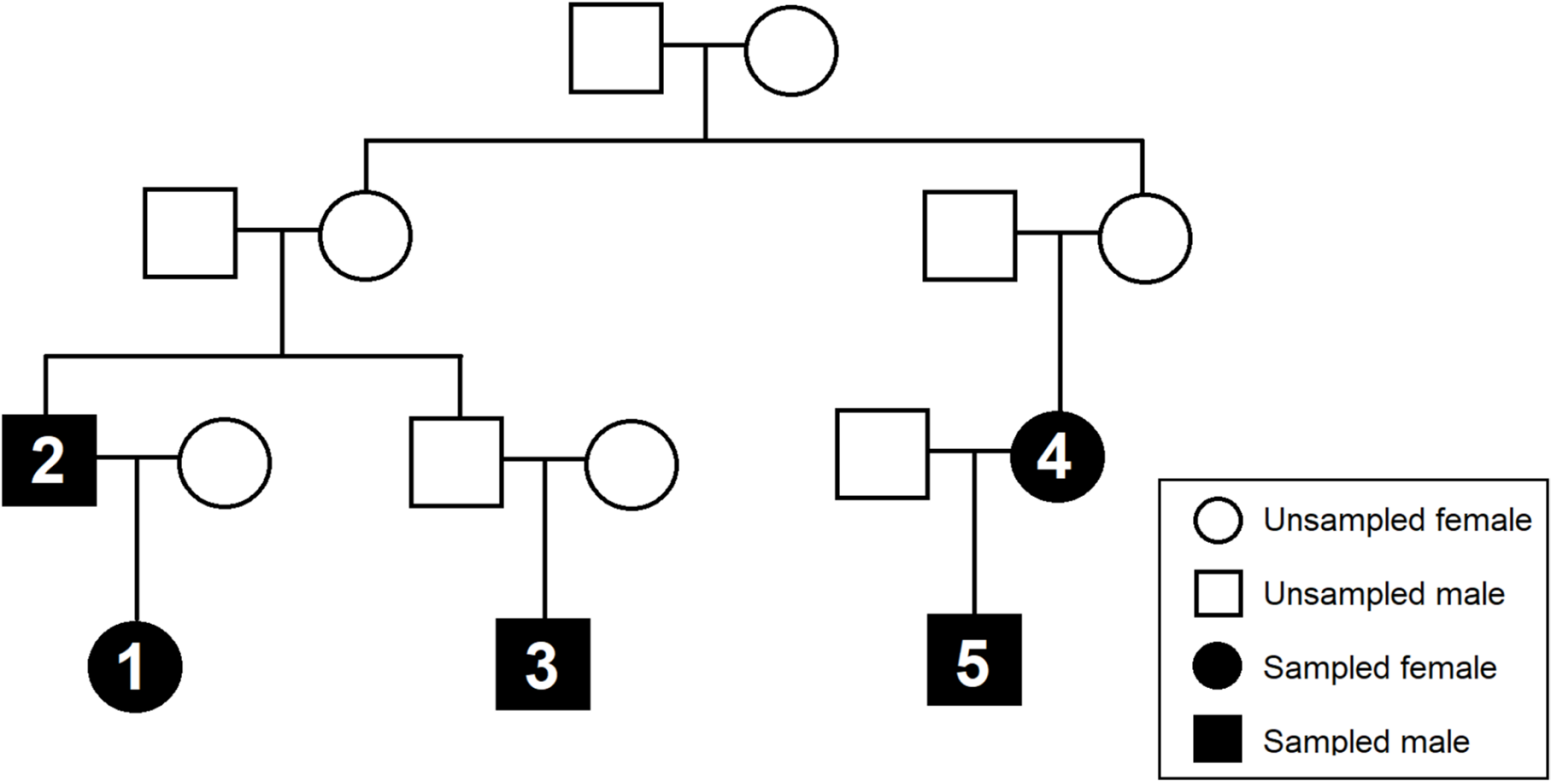
Pedigree chart of the genetically related volunteers; individuals sampled are filled in (black) and marked 1 through 5, representing samples Family 1 through Family 5

Reference-type samples included buccal swabs collected from genetically related volunteers spanning 1 st to 5 th degree relationships (n = 5, Fig. 1). Casework-type samples included bone (n = 2), tooth (n = 2) and nail (n = 1) samples sourced from AFTER, volunteers and approved research and casework samples submitted to the AFP National DNA Program for Unidentified and Missing Persons. The positive control (PC) DNA sample was NA24385 lymphoblastoid cell line (provided with the Kintelligence Kit) [16] and the negative control (NC) was nuclease-free water.

DNA was extracted from buccal swabs and nail samples using the QIAamp DNA Investigator® Kit (QIAGEN, Hilden, Germany) [26]. For bone and tooth samples, 500 mg of pulverised bone or tooth powder underwent total demineralisation lysis, concentration using an Amicon® 30 K Ultra Centrifugal Filter (Sigma-Aldrich, St. Louis, MO, US) and purification with the MinElute® PCR Purification Kit (QIAGEN) [27–29]. All samples were quantified using the Quantifiler™ Trio DNA Quantification Kit (Thermo Fisher Scientific, Waltham, MA, US) on a QuantStudio™ 5 Real-Time PCR System (Thermo Fisher Scientific) according to the manufacturer’s protocol [30, 31]. The PC (NA24385) was quantified with the QuantiFluor® ONE dsDNA System (Promega, Madison, WI, US) on the Quantus™ Fluorometer (Promega). [32, 33]

### Library Preparation, Sequencing and Bioinformatics

**Fig. 2:**
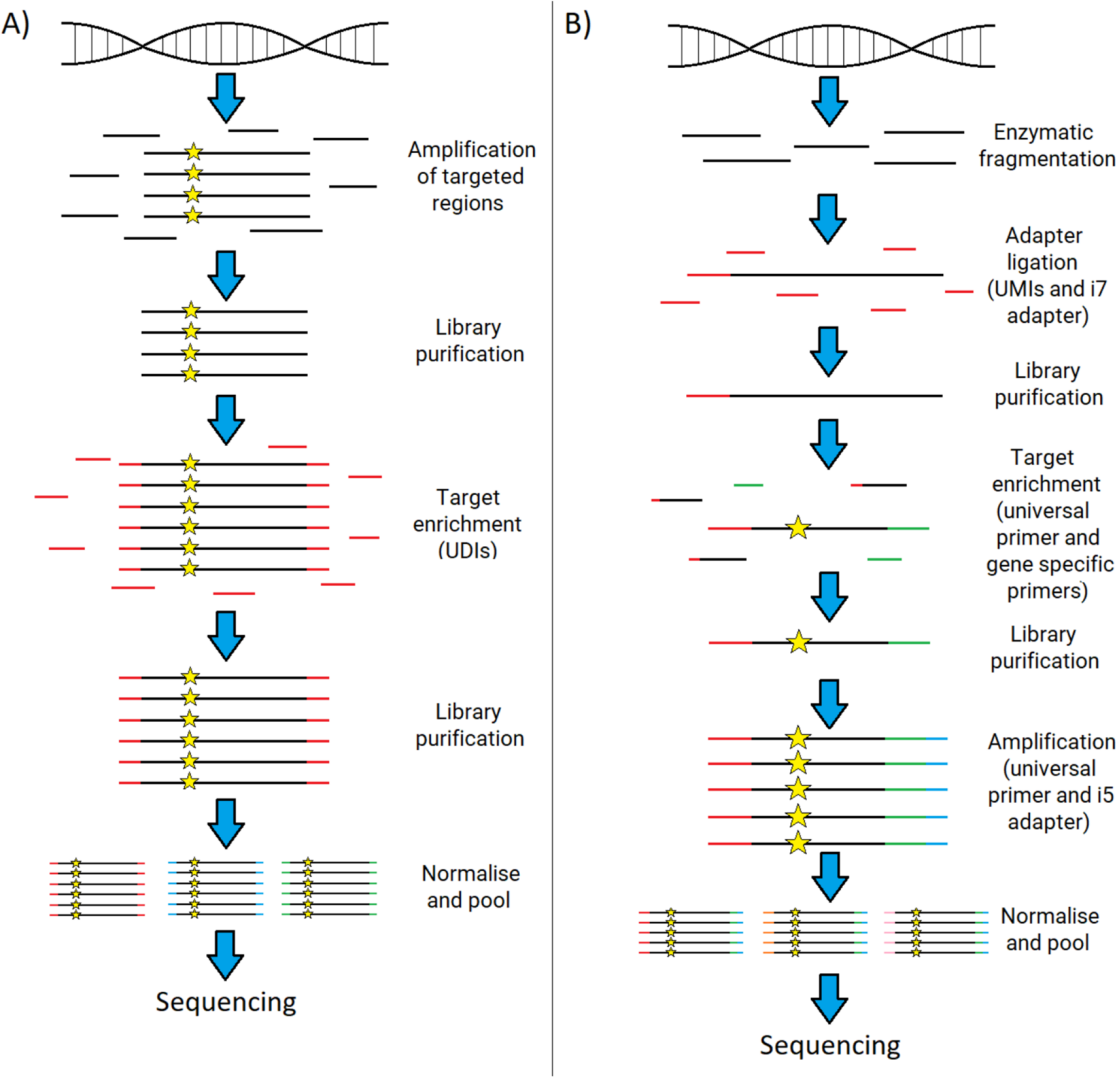
Library preparation workflows for the A) ForenSeq® Kintelligence Kit and B) FORensic Capture Enrichment (FORCE) panel with the QIAseq workflow. The star represents the SNP being targeted. [16, 17, 34, 35]

For all pipelines, the DNA input amount was calculated from the large autosomal (LA) target concentration, to avoid overdiluting degraded samples. Extracted DNA was then diluted accordingly with nuclease-free water. Table 2 outlines the quantification results, degradation index (DI) and the DNA input calculated for the Kintelligence Kit (maximum of 1 ng in 25 µL) and the FORCE panel (maximum of 10 ng in 18.43 µL) workflows. The pipelines for library preparation are outlined in Fig. 2. The Veriti™ 96-Well Fast Thermal Cycler (Thermo Fisher Scientific) was used for the Kintelligence and FORCE pipelines.

**Table 2:** Sample DNA concentrations, degradation indices and calculated input amount for the ForenSeq® Kintelligence Kit and FORensic Capture Enrichment (FORCE) panel

Sample ID	Sample Type	Large Autosomal (LA) Target (ng/µL)	Degradation Index (DI)	DNA Input Amount (ng)	
				ForenSeq® Kintelligence Kit	FORCE Panel
NA24385	Control DNA	10.000 ^a^	N/A	1.00	10.00
Family 1	Buccal	0.006	3.33	0.15	0.11
Family 2	Buccal	0.037	1.15	0.94	0.69
Family 3	Buccal	0.203	1.46	1.00	3.74
Family 4	Buccal	0.095	2.53	1.00	1.76
Family 5	Buccal	0.058	1.14	1.00	1.06
Tooth 1	Tooth	0.007	5.49	0.17	0.12
Tooth 2	Tooth	0.379	1.08	1.00	6.99
Bone 1	Bone	0.322	1.01	1.00	5.94
Bone 2	Bone	0.009	82.94	0.74	0.16
Nail 1	Nail	0.747	1.26	1.00	10.00

^a^ Concentration of double stranded DNA in solution

#### ForenSeq® Kintelligence Kit

The libraries were prepared manually in batches of 10 samples together with a PC (NA24385) and NC [36]. The amplified products were purified and barcoded using unique dual indices (UDIs), before being individually normalised to 0.75 ng/µL using the QuantiFluor® ONE dsDNA System (Promega, Madison, WI, US) on the Quantus™ Fluorometer (Promega) [32, 33]. The normalised libraries were then pooled in batches of three samples. The PC and NC were included in the first pool only. Sequencing of each pool was performed on the MiSeq FGx® Sequencing System with the standard flow cell (SFC) [37]. Different UDI combinations were used on subsequent runs to limit the effect of sample carryover.

The sequencing run metrics were assessed using the Universal Analysis Software (UAS) v2.5 (Verogen, Inc.) and the Sequencing Analysis Viewer (SAV; Illumina, San Diego, CA, US) for cluster density, clusters passing filter, reads passing the quality score of 30 (Q30) threshold and approximation of adapter dimers present on the SFC [38, 39]. The genotypes were exported from the UAS and analysed according to the optimised thresholds and Microsoft Excel macro workbook previously published by Watson et al*.* (2023) to generate the final genotype [36]. This included a total read threshold of 20 reads and relative allele frequency thresholds for homozygotes (0.95–1.00), heterozygotes (0.10–0.90), sequencing error (< 0.10) and ambiguous variants (with relative frequencies in the ranges 0.05–0.10 and 0.90–0.95).

#### FORensic Capture Enrichment Panel

The FORCE panel library preparation followed the single primer extension workflow specified in the May 2017 version of the QIAseq Targeted DNA Panel handbook unless otherwise stated [34]. All reagent volumes were based on the standard DNA option and increased by 10% for all parts of the workflow to allow for transferring the libraries between 96-well semi-skirted and midi plates for the purifications. The libraries were prepared manually in a batch of 24 samples together with a PC (NA24385) and NC.

Fragmented DNA was tagged with unique molecular indices (UMIs) and i7 adapters from the QIAseq 12-index I set of combinatorial dual index (CDI) adapters. The targets were purified, enriched using the universal primer and gene specific primers and amplified after being tagged with the universal primer and i5 adapter. Libraries were normalised to 1.00 ng/µL using the QuantiFluor® ONE dsDNA System on the Quantus™ Fluorometer. The normalised libraries were then pooled in batches of six samples. Sequencing of each pool was performed on the MiSeq FGx® Sequencing System with the SFC in “Research Use Only” mode. [37]

The sequencing run metrics were assessed using the SAV for cluster density, clusters passing filter, reads passing the Q30 threshold and approximation of adapter dimers present on the SFC. FASTQ files were exported from the MiSeq FGx® Sequencing System, imported into the CLC Genomics Workbench v22.0.2 (QIAGEN) and analysed using a custom workflow published by Tillmar et al*.* (2021). [15, 40] The FORCE genotypes were exported in CSV files and additional relative allele frequency thresholds were applied for homozygotes (0.90–1.00), heterozygotes (0.20–0.80), sequencing error (< 0.10) and ambiguous variants (with relative frequencies in the ranges 0.10–0.20 and 0.80–0.90).

### Genotype Analysis

For the SNPs that were shared between the Kintelligence Kit and FORCE panel (Table 1), the genotypes were analysed for coverage, call rates, autosomal heterozygosity and genotype concordance. Differences in the genotypes that could not be explained by sequencing on the opposite strand were classified as non-concordant SNPs.

### Phenotype and Ancestry Estimation

For the Kintelligence genotypes, the reported phenotypes and ancestries were exported from the UAS. This included the probabilities for hair colours (blond, brown, red and black) and eye colours (brown, blue and intermediate) using an embedded multinomial logistic regression (MLR) algorithm. An in-built principal component analysis (PCA) is used to infer BGA from four population clusters (European, East Asian, African and Admixed American).

Text files of the FORCE genotypes were imported to FamLink2 for analysis with the Phenotype/Ancestry/Haplogroup tool [21]. The FORCE panel genetic map and allele frequencies for several populations (African, American, East Asian, European, Middle Eastern, Oceanian and South Asian) were sourced from the FamLink2 database and imported into FamLink2 for BGA inference [41]. A CSV file was exported from FamLink2 for upload to HIrisPlex-S to infer hair colour (blond, brown, red or black), hair shade (light or dark), eye colour (brown, blue or intermediate) and skin colour (very pale, pale, intermediate, dark or dark-to-black). [42–44]

### Kinship Analysis

To calculate the LR for kinship analysis, the conditional probabilities of observing the genotypes given two alternative propositions were compared for each pairwise combination of the five genetically related individuals. The first proposition corresponded with the true relationship between the individuals and the second proposed that they were unrelated. For example:

*H*_*1*_*:* The donor of profile 1 and the donor of profile 2 are full siblings.

*H*_*2*_*:* The donor of profile 1 and the donor of profile 2 are unrelated members of the European population.

LRs were calculated using DBLR™ v1.3 (STRmix™) [45]. The genetic linkage map and European population allele frequency data were downloaded from the FamLink2 database [41]. The Kintelligence Kit map was constructed using the Map Interpolator of the Rutger’s Map v.3 with the sex-average centimorgan (cM) positions [46]. The allele frequencies for the autosomal SNPs targeted in the Kintelligence Kit were sourced from the 1000 Genomes Project for a European population. [47]

## Results and Discussion

### Sequencing Performance

For the Kintelligence Kit pipeline, the 12 samples were distributed over five sequencing runs in pools of three libraries. The majority of the sequencing metrics for the runs were within the recommended ranges for sequencing on the MiSeq FGx® Sequencing System; cluster density was slightly higher than the recommended range for one sequencing run (Table 3) [37, 38]. The higher densities of the Kintelligence runs correlated with lower percentages of adapter dimers present on the SFC.

**Table 3:** Performance metrics for the ForenSeq® Kintelligence Kit (n = 5) and FORensic Capture Enrichment (FORCE) panel (n = 3) sequencing runs

Metric	ForenSeq® Kintelligence Kit	FORCE Panel
Cluster Density (K/mm^2^)	1037–1893	199–451
Clusters Passing Filter (%)	75.0–89.9	91.0–93.5
Reads Passing Q30^a^ (%)	63.4–85.4	81.7–84.5
Adapter Dimers (%)	11–27	65–70
Libraries per SFC	3	6

^a^ Quality score of 30 where there is an error rate of 1 in 1000, with a corresponding call accuracy of 99.9%

For the FORCE pipeline, the samples were sequenced in batches of six libraries over four sequencing runs. The first sequencing run failed, which included the Family 1 reference-type sample, and was excluded from analysis. The FORCE runs had lower cluster densities and a greater presence of adapter dimers compared to the Kintelligence runs (Table 3). However, the clusters passing filter and reads passing the Q30 were, on average, higher than the Kintelligence runs.

An evaluation of 15 sequencing runs with the FORCE QIAseq workflow by Staadig et al*.* (2023) revealed a significantly higher average cluster density (1032 K/mm^2^ ± 318 K/mm^2^), with 89% of reads passing the Q30 threshold [17]. The number of libraries pooled by Staadig et al*.* (2023) varied from 3 to 8 libraries per pool with a DNA input of 1 to 10 ng, as opposed to 6 libraries with DNA input ranging from 0.11 to 10 ng in this study. The low DNA inputs of the samples tested in this study likely resulted in the low cluster density of the sequencing runs despite the similar sequencing plexity. The large proportion of adapter dimers in the FORCE runs may also result from the reduced amount of template DNA for the samples that were below the recommended DNA input of 10ng, resulting in higher concentrations of adapters and primers, facilitating formation of dimers.

### Quality and Quantity of the Genetic Information

**Fig. 3:**
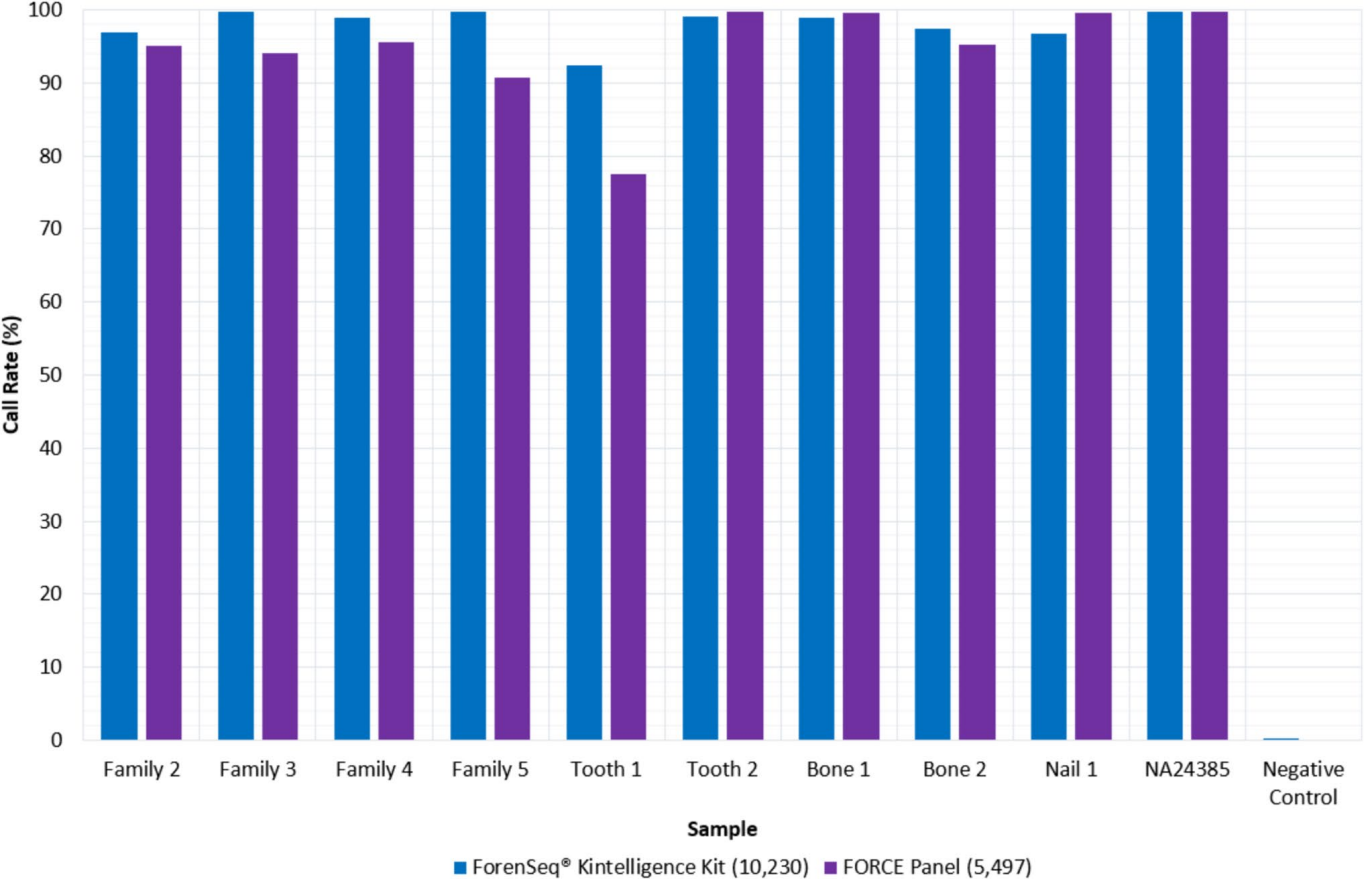
Sample call rates for sequencing with the ForenSeq® Kintelligence Kit and FORensic Capture Enrichment (FORCE) panel

Of the ten Kintelligence genotypes, 70% of samples met the recommended DNA input amount of 1.0 ng (Table 2) and at least 92% of loci were called (Fig. 3). There was no discernible impact of low DNA inputs on the average call rate (98.0% ± 2.2%) or autosomal heterozygosity (47.4% ± 0.7%) for the samples. Previous evaluations of the Kintelligence Kit have demonstrated that it is able to produce high quality SNP genotypes from low template amounts, particularly for sample types commonly encountered in UHR casework [13, 36, 48]. The NC had two SNPs called and this is in line with the rate of sequencing error observed in NCs from other studies and unlikely to be the result of contamination. [13, 36, 48]

Only 20% of the samples (NA24385 and Nail 1) met the 10.0 ng DNA input amount recommended for the FORCE panel (Table 2). Only one sample produced a call rate below 94%; Tooth 1 had a low DNA input amount of 0.12 ng and call rate of 77.5% (Fig. 3). Similar to the Kintelligence genotypes, there was no discernible impact of low DNA inputs on the average call rate (94.7% ± 6.4%) or autosomal heterozygosity (33.6% ± 7.2%). The FORCE NC had no SNPs called.

Both panels produced high quality genotypes for Bone 2 despite the severe degradation (DI = 83, Kintelligence call rate = 96.9%, FORCE call rate = 95.0%). This is consistent with the previous observations from validations of both pipelines [13, 36, 48]. In the developmental validation of the Kintelligence Kit, 69% (11) of bone samples – including burned, cremated, embalmed, buried and ancient bones – produced profiles with call rates over 90% [13]. Dilutions of the buried bone samples improved the sequencing performance, indicating that inhibitors were present. The internal validation of the Kintelligence Kit by Peck et al*.* (2022) reported 64% (7) of bone samples with DNA inputs ranging from 0.1 to 1.0 ng produced call rates of at least 96.9% [48]. Furthermore, call rates exceeding 90% were obtained for a range of UHR casework-type samples tested by the AFP National DNA Program for Unidentified and Missing Persons as part of their Kintelligence Kit validation. [36]

Similarly, the FORCE panel was designed for sequencing poor quality samples with severely degraded DNA; initially using hybridisation capture to target DNA fragments less than 75 bp long [15]. When testing 12 bone samples using different DNA extraction methods, an average of 44.4% of SNPs were called during the developmental validation [15]. An evaluation of the FORCE panel’s QIAseq workflow using 1.0 ng of input DNA for testing bone and tissue samples revealed an average call rate of 97.6% and 90.7% of SNPs, respectively. [17]

The 992 SNPs in common between the Kintelligence Kit and FORCE panel were assessed for call rate, autosomal heterozygosity and genotype concordance (Table 4). The Kintelligence genotypes produced higher call rates than the FORCE panel for 7 of the 10 samples; however, there were no substantial deviations with the exception of Tooth 1. The Kintelligence Kit was less susceptible to locus dropout of aiSNPs, iiSNPs and kiSNPs, returning higher call rates than the FORCE panel for these SNP classes. The FORCE panel recovered the piSNPs with greater consistency. When assessing the autosomal SNPs (979), the average heterozygosity was 45.2% ± 2.2% for Kintelligence and 39.8% ± 7.8% for FORCE genotypes. It is likely there was substantial allele dropout in the FORCE genotypes for Tooth 1, as only 19.5% of autosomal SNPs were heterozygous.

**Table 4:** Call rate, autosomal heterozygosity and concordance for the 992 SNPs in common between the ForenSeq® Kintelligence Kit and FORensic Capture Enrichment (FORCE) panel

Sample ID	Biological Sex	Call Rate (%)		Autosomal Heterozygosity (%)		Concordance (%)
		ForenSeq® Kintelligence Kit	FORCE Panel	ForenSeq® Kintelligence Kit	FORCE Panel	
Family 2	Male	97.5	94.4	43.5	41.1	89.4
Family 3	Male	99.9	93.3	47.0	38.7	86.6
Family 4	Female	98.7	93.6	45.0	38.2	86.9
Family 5	Male	99.8	92.8	49.5	34.3	78.6
Tooth 1	Female	91.6	77.7	42.9	19.5	51.5
Tooth 2	Female	98.8	98.3	45.4	45.8	97.5
Bone 1	Male	99.2	99.5	47.7	48.3	97.7
Bone 2	Male	96.9	95.0	43.4	42.6	88.8
Nail 1	Male	96.4	99.6	42.2	44.8	94.3
NA24385	Male	99.6	99.6	44.9	44.6	98.0

**Fig. 4:**
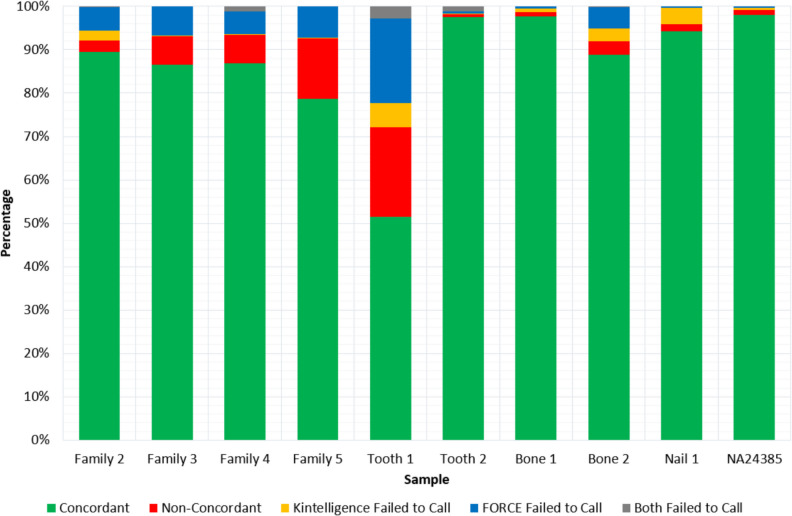
Distribution of concordance, non-concordance and no-calls for the 992 SNPs in common between the ForenSeq® Kintelligence Kit and FORensic Capture Enrichment (FORCE) panel

The average concordance between the Kintelligence and FORCE genotypes was 86.9% ± 13.2%, with the greatest concordance observed with the casework-type samples (Fig. 4). Tooth 1 had the lowest concordance rate of 51.5%, whereas the control DNA NA24385 had the highest at 98.0%. When compared to the known Kintelligence genotype for NA24385, both Kintelligence and FORCE had a genotype concordance of 99.6% [38]. Across the ten samples, the average non-concordance rate was 5.8% ± 6.3%, ranging from 0.7% (Tooth 2) to 20.7% (Tooth 1).

**Fig. 5:**
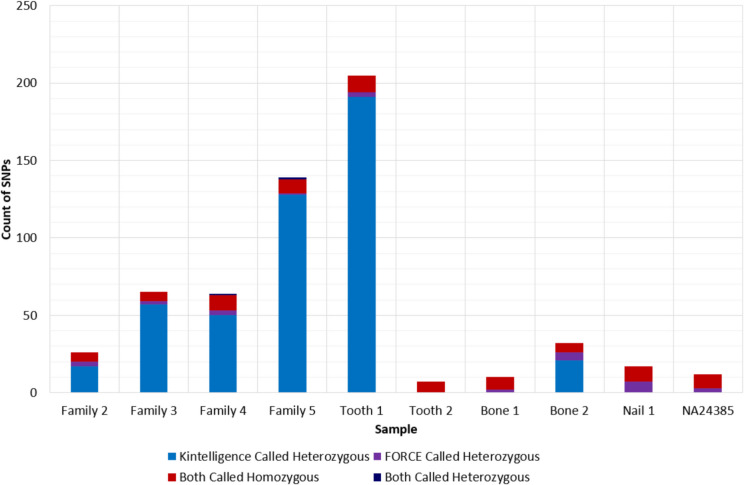
Types of non-concordant SNPs observed in each sample between the ForenSeq® Kintelligence Kit and FORensic Capture Enrichment (FORCE) panel

Of the 573 non-concordant SNPs observed over the 10 samples, 81% of SNPs were called as heterozygous by the Kintelligence Kit and homozygous for only one of those alleles by the FORCE panel, suggesting a high incidence of allele dropout for the FORCE panel (Fig. 5). Conversely, only 5% of non-concordant SNPs were called as heterozygous in the FORCE genotypes. The remaining non-concordance could not be explained by sequencing on the opposite strand; 14% were called homozygous for different alleles by both pipelines and only two kiSNPs were called as heterozygous by both panels. These two kiSNPs exhibited very low coverage when sequenced with the FORCE panel. They were rs2706586 for Family 4 (Kintelligence typed GT from 2312 reads; FORCE typed CG from 3 reads) and rs4660390 for Family 5 (Kintelligence typed CT from 160 reads; FORCE typed CG from 3 reads). There were no discernible trends in the non-concordance or locus dropout by SNP class except for rs1805005, a piSNP on the MC1R gene. In the eight samples where both pipelines called this SNP, the FORCE genotype was TT for all samples and the Kintelligence genotype was either GG or TT.

When comparing the read data produced for the 992 overlapping SNPs, the libraries prepared with the Kintelligence Kit returned significantly higher total sequencing reads per sample than those prepared with the FORCE panel (p < 0.01; Table 5). Furthermore, the median reads per SNP was significantly higher for the Kintelligence genotypes (p < 0.001). It is important to note, however, that the FORCE reads are unique reads, each one corresponding with a unique UMI. The higher median reads per SNP for the Kintelligence Kit include PCR duplicates which are combined into unique reads for the FORCE panel.

**Table 5:** Total sequencing reads and median reads per SNP for just the 992 overlapping SNPs for each sample sequenced with the ForenSeq® Kintelligence Kit and FORensic Capture Enrichment (FORCE) panel

Sample ID	ForenSeq® Kintelligence Kit		FORCE Panel	
	Total Sequencing Reads	Median Reads per SNP	Total Sequencing Reads	Median Reads per SNP
Family 2	638,043	432	8,623	8
Family 3	1,583,992	1,047	6,916	6
Family 4	1,126,906	783	8,797	8
Family 5	836,849	569	4,282	4
Tooth 1	323,234	214	1,937	2
Tooth 2	1,531,494	1,047	43,823	44
Bone 1	372,427	268	46,174	45
Bone 2	2,369,358	1,425	14,383	13
Nail 1	225,068	151	52,839	51
NA24385	1,691,205	1,163	72,406	71

### Phenotype, Ancestry and Kinship Inferences

Table 6 outlines the EVC and BGA inferences generated using the Kintelligence Kit and FORCE panel analytical pipelines against the self-declared data provided by the volunteers. The Kintelligence genotype for Tooth 1 did not yield all 24 piSNPs and, subsequently, the embedded MLR algorithm was unable to generate p-values for hair and eye colour inference.

**Table 6:** Phenotype and ancestry inferences based on the highest p-values for the genotypes produced with the ForenSeq® Kintelligence Kit and FORensic Capture Enrichment (FORCE) panel compared to the self-declared phenotype and ancestry

Sample	ForenSeq® Kintelligence Kit	FORCE Panel	Self-Declared
Family 2	Blue eye colour (0.91) Brown hair colour (0.83) European ancestry	Blue eye colour (0.90) Brown hair colour (0.81) Light hair shade (0.82) Pale skin colour (0.75) European ancestry	Grey eye colour Brown hair colour Pale skin colour European ancestry
Family 3	Brown eye colour (0.83) Brown hair colour (0.64) European ancestry	Brown eye colour (0.84) Brown hair colour (0.73) Light hair shade (0.75) Intermediate skin colour (0.79) European ancestry	Brown eye colour Brown hair colour Intermediate skin colour European ancestry
Family 4	Blue eye colour (0.89) Blond hair colour (0.73) European ancestry	Blue eye colour (0.88) Red hair colour (0.49) Light hair shade (0.99) Pale skin colour (0.70) European ancestry	Green eye colour Brown hair colour Intermediate skin colour European ancestry
Family 5	Blue eye colour (0.94) Blond hair colour (0.78) European ancestry	Blue eye colour (0.93) Red hair colour (0.47) Light hair shade (1.00) Pale skin colour (0.75) European ancestry	Blue eye colour Red hair colour Very pale skin colour European ancestry
Tooth 1	European ancestry	Brown eye colour (0.40) Brown hair colour (0.84) Dark hair shade (0.88) Pale skin colour (0.42) European ancestry	Hazel eye colour Brown hair colour Pale skin colour European ancestry
Tooth 2	Blue eye colour (0.92) Blond hair colour (0.88) European ancestry	Blue eye colour (0.91) Blond hair colour (0.71) Light hair colour (1.00) Pale skin colour (0.64) European ancestry	Blue eye colour Blond hair colour Intermediate skin colour European ancestry

All BGA inferences for both workflows were consistent with the self-declared ancestry (European in all cases). For the UAS, all Kintelligence aiSNP genotypes were exclusively within the European cluster on the PCA plot, indicating the genotypes were more consistent with European ancestry than East Asian or African. It has been observed in previous studies that the PCA method on the UAS is limited by the small number of available populations [36]. As the volunteers in this study all self-declared European ancestry, this was not problematic for our study. However, this limitation could impact the inference of ancestries for individuals outside of the European, Asian and African populations, as well as for the interpretation of admixture. [36, 49, 50]

FamLink2 uses a naïve Bayesian approach to estimate the likelihood probability for each reference population [21]. All FORCE genotypes were more likely to be of European ancestry than African, American, East Asian, Middle Eastern, Oceanic or South Asian ancestry. It has previously been demonstrated that BGA inferences derived from the FORCE panel using this pipeline were consistent with self-declared ancestry for nine genotypes of European, Hispanic and African American ancestry. [15, 17]

The eye, hair and skin colour inferences presented in Table 6 are based on the highest p-value obtained from the Kintelligence and FORCE analytical pipelines. All eye colour inferences were consistent across both pipelines, indicating these individuals were more likely to have brown or blue eyes. There were two inferences that were inconsistent with the self-declared data. The first individual, Family 4, self-declared having intermediate (green) eye colour and the p-values for both panels were highest for blue eye colour. The second individual, Tooth 1, self-declared intermediate (hazel) eye colour and only the FORCE panel was able to generate p-values, which were highest for brown eye colour. Previous EVC studies have shown that the HIrisPlex algorithm has lower success rates for inferring intermediate eye colours [36, 51, 52]. For the Kintelligence Kit, all inconsistent eye colour inferences corresponded with individuals who had self-declared intermediate eye colours [36]. In the development of the FORCE panel, the sole volunteer who self-declared an intermediate eye colour was inferred to have blue eyes. [15]

Hair colour inferences were consistent between analytical pipelines and the self-declared data for three of the genotypes (Table 6). The inference generated with the FORCE genotype for Tooth 1 was also consistent with the self-declared data, whereas analysis of the Kintelligence genotype was unable to produce p-values without a full piSNP profile. The panels were inconsistent with each other for inferring the hair colour for Family 4 and Family 5. Family 5 has self-declared red hair and was inferred as red with the FORCE pipeline but inferred as blond with the Kintelligence pipeline. Further interrogation of the Kintelligence SNP data showed that there was alignment ambiguity at N29 insA (also denoted rs312262906) on the MC1R gene from position 16:89,919,340 to 16:89,919,344 leading to a heterozygous SNP (A/C) being called at 16:189,919,342 instead of a heterozygous A insertion at 16:89,919,344. This is one of the SNPs used to infer red hair colour [53]. The piSNP genotype was analysed using the online HIrisPlex tool to determine the impact of this alignment ambiguity [42, 44, 54]. The Kintelligence genotype called NA29 insA as homozygous C and produced a p-value of 0.111 for red hair colour; when the genotype was adjusted to a heterozygous A insertion, the p-value for red hair colour increased to 0.997.

Skin colour inferences were only possible with the FORCE genotypes as the panel included the additional 17 piSNPs in the HIrisPlex-S panel [42–44]. The largest p-values were for pale and intermediate skin colours, and these were largely consistent with the self-declared skin colours. Skin colour can be complex to infer depending on population-specific influences and environmental factors. [42, 43]

**Fig. 6:**
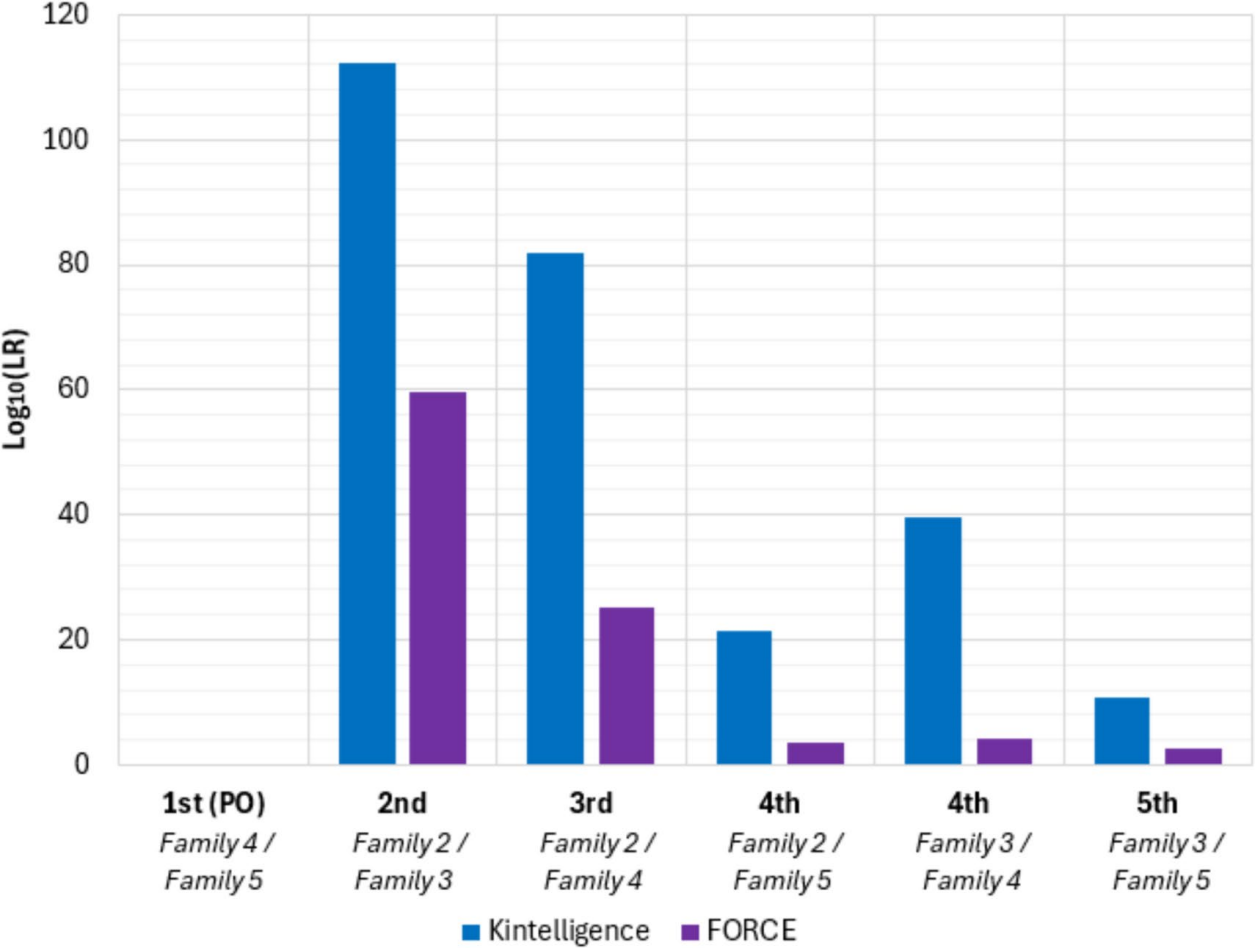
Logarithms of the likelihood ratios (LR) generated for six pair-wise combinations of pedigree members using the ForenSeq.® Kintelligence Kit and FORCE panel. LRs were unable to be calculated for the 1 st degree relationship between Family 4 and Family 5 (parent/offspring (PO))

LRs were calculated for six pair-wise combinations of the four related volunteers using the full autosomal SNP potential of each panel (Family 1 was excluded). As a result of the Kintelligence Kit targeting nearly twice as many SNPs than the FORCE panel, it was expected that the LRs would be significantly higher (Fig. 6). The LR for the true parent/offspring relationship (1 st degree) was unable to be calculated using DBLR™ as there was not an allele in common at every locus. Due to the high density of these panels, it is likely that at least one instance of allele dropout will occur in samples such as the buccal swabs collected from volunteers in this study.

The LRs were generated for each of the relationship pairs and for each of the Kintelligence and FORCE genotypes to test the 2nd through 5 th degree relationships. According to the forensic verbal equivalency scale, the LRs for all Kintelligence relationships and FORCE 2nd and 3rd degree relationships provided “very strong support” (LR > 1,000,000) [55]. For the remaining FORCE genotype pairings, 4 th degree relationship LRs provided “moderately strong support” (LR > 1,000) and the 5 th degree relationship LR provided “moderate support” (LR > 100).

Gettings et al*.* (2024) compared the Kintelligence Kit and FORCE panel for disaster victim identification (DVI) applications and evaluated kinship predictions using data simulations for 1 st to 5 th degree relationships using the iiSNPs and kiSNPs available (9,959 SNPs for the Kintelligence Kit and 4,073 SNPs for the FORCE panel) [56]. They observed the median log_10_ LR decreased by a factor of three for each increase in relationship degree. The log_10_ LRs calculated for the samples in this study for all autosomal SNPs (10,039 SNPs for the Kintelligence Kit and 4,368 SNPs for the FORCE panel) were consistent with those published by Gettings et al*.* for 2nd to 5 th degree relationships [56]. However, LRs were unable to be generated in this study for 1 st degree relationships (parent/offspring) due to allele dropout, whereas Gettings et al*.* generated median log_10_ LRs of 1300 for these relationships using simulated samples with no allele dropout. [56]

### Considerations for Implementation

There are several advantages to the implementation of medium-density TAS SNP panels. As these pipelines can be operationalised with existing MPS workflows and instrumentation, the entire laboratory and analytical process can be performed in-house, maintaining control over the chain of custody and quality processes [3, 57, 58]. Table 7 summarises the requirements for implementing both TAS pipelines.

**Table 7:** Facility, sample and bioinformatics requirements and generatable genetic intelligence for the ForenSeq® Kintelligence Kit and FORensic Capture Enrichment (FORCE) panel with the QIAseq workflow

Component	ForenSeq® Kintelligence Kit	FORCE Panel
TAS Workflow	PCR amplicon-based	Single primer extension
Library Preparation Cost (USD)^a^	$15,720 [56]	$1,745 (QIAseq Kit) [38] $1,580 (Index Kit) [69]
Sequencing Cost (USD)^b^	$2,085 [56]	$2,085 [56]
**Sample Requirements**		
Input Volume	25 µL	16.75 µL
Recommended DNA Input	≥ 1 ng	≥ 10 ng
Sample Quality	Can be severely degraded	Can be severely degraded
Recommended Library Preparation Plexity	12	48 (antemortem) 16 (postmortem) [56]
Recommended Sequencing Plexity	3	48 (antemortem) 16 (postmortem) [56]
**Genetic Intelligence**		
Bioinformatics Required	No	Yes
Individual Identification	Yes	Yes
Y Haplogroup Prediction	Yes	Yes
X Chromosome Analysis	Yes	Yes
Externally Visible Characteristics Inference	Yes; hair and eye colour	Yes; hair, eye and skin colour
Biogeographical Ancestry Inference	Yes	Yes
Extended Kinship Analysis	Yes; 1 st to 5 th degree relatives	Yes; 1 st to 5 th degree relatives
Genetic Genealogy Database Compatibility	GEDmatch PRO™ and FamilyTreeDNA	No

^a^ Kit component costs calculated for 12 libraries included in the Kintelligence and FORCE QIAseq Kits

^b^ Cost of MiSeq FGx® Reagent Kit for operation on the MiSeq FGx® Sequencing System

#### Facility Requirements

Both TAS pipelines require similar consumables and storage requirements for the components for library preparation and sequencing. Furthermore, similar laboratory skills are required such as the handling of magnetic beads for library purification. The differences between the TAS workflows are most discernible in the setup of pre-amplification and post-amplification laboratory spaces, as the FORCE panel requires use of a thermocycler for adapter ligation and target enrichment before the DNA is amplified, requiring equipment typically housed in the post-amplification space to be accessible in the pre-amplification space. [34]

For incorporation into a laboratory environment, both TAS workflows are compatible with sequencing on the MiSeq FGx® Sequencing System which has a user-friendly interface and generates exportable data in a FASTQ format. The advantage of using a benchtop sequencer and MPS technology is that more forensic laboratories are expressing interest in pursuing MPS capabilities and acquiring this instrumentation [59, 60]. A 2023 study of US forensic laboratories revealed 163 facilities already have a MiSeq FGx® Sequencing System for forensic genomics applications. [60]

Routine STR typing employed by forensic biology laboratories for human identification is relatively inexpensive compared to emerging technologies. For example, the GlobalFiler™ PCR Amplification Kit (Thermo Fisher Scientific) costs US$4,350 for autosomal STR typing of 200 samples [61]. At the time of publication, the cost of the Kintelligence Kit (US$15,720) was greater than the components required for FORCE (US$3,325) and both kits have enough reagents to prepare 12 libraries [62, 63]. The MiSeq FGx® Reagent Kit (Verogen, Inc.) required for sequencing costs US$2,085, but the cost per library is dependent on the sequencing plexity validated by the laboratory. [64]

The recently released ForenSeq® Kintelligence HT Kit offers a higher throughput workflow, reducing the cost per sample by increasing the sequencing plexity to up to 12 postmortem and 36 antemortem samples per SFC [65]. At the time of writing, the kit contains reagents for 96 samples at a cost of approximately US$88,800 [66]. However, as sequencing plexity increases, the number of SNPs typed deceases, which limits the downstream applications. For example, to perform FIGG, at least 70% of Kintelligence SNPs are required for upload to the law enforcement genetic genealogy databases. [67]

#### Sample Requirements

Forensic laboratories seeking to operationalise an in-house TAS pipeline will need to consider the typical sample types and conditions encountered in casework. The amount of DNA recovered from UHR can vary due to differences in DNA preservation rates, degradation from environmental exposure and the postmortem interval [1]. The TAS panels tested in this study have been specifically designed for forensic applications and have demonstrated broad suitability for samples commonly encountered in UHR and missing persons cases. Both pipelines demonstrated suitability for reference- and casework-type samples, including bone, tooth, nail and buccal samples. The success with an array of compromised samples aligns with findings from previous studies. [13, 15, 17, 36, 48]

Furthermore, while manufacturer protocols recommended DNA inputs of 1.0 ng and 10.0 ng for Kintelligence and FORCE library preparations, respectively, previous sensitivity studies have observed high quality genotypes for lower input amounts [13, 17, 36, 48]. In this study, samples with inputs as little as 0.12 ng still produced nearly complete Kintelligence genotypes suitable for further analysis. However, the performance of FORCE was negatively impacted by the lower than recommended DNA inputs in this study, resulting in comparatively high numbers of adapter dimers and low cluster density for the sequencing runs and allele dropout in the FORCE genotypes. This is important, as DNA input might be limited by the volume of DNA extract remaining for a sample, especially since SNP genotyping is typically conducted after STR typing. [68]

#### Bioinformatics Requirements and Genetic Intelligence Applications

The Kintelligence and FORCE analytical pipelines differ substantially in their methods for producing genotypes and associated genetic intelligence. The UAS circumvents the need for an external bioinformatics system when sequencing with the Kintelligence Kit by integrating multiple bioinformatic processes to generate a final genotype from raw sequencing data, infer hair and eye colour using an MLR algorithm and infer BGA using PCA [13, 38]. This streamlined approach is presented through a user-friendly interface that requires no prior bioinformatics knowledge or experience to operate and any additional analyses performed in this study for genotype generation utilised Microsoft Excel. With the UAS v2.7 update, the piSNP genotype can be exported and uploaded to the HIrisPlex website for hair and eye colour inference [69]. The HIrisPlex tool calculates area under the curve values to estimate the impact of locus dropout on the generated p-values. [42–44]

In contrast, the FORCE workflow requires additional bioinformatics systems for analysing the FASTQ files generated by the MiSeq FGx® Sequencing System in “Research Use Only” mode. Such systems, including the CLC Genomics Workbench used in this study, incur additional costs to the laboratory [40]. Although the FORCE panel targets approximately half the number of SNPs compared to the Kintelligence Kit, it includes a greater number of non-kinship SNPs, enabling expanded genetic intelligence, including skin colour inference, higher resolution BGA inference and Y haplogroup prediction [15]. The FamLink2 software, a freely available application, allows for further analysis of the finalised genotype [21]. This includes the comparison of the questioned genotype to population data using a naive Bayesian approach for BGA inference, the production of probabilities for hair and eye colours and the capability to generate an input file for upload to the HIrisPlex-S website. [42–44]

Both TAS pipelines were able to produce genetic intelligence for hair colour, eye colour and BGA, as well as skin colour for the FORCE panel, that were largely consistent with the self-declared data provided by the volunteers. While both panels generated X and Y SNP genotypes, these were not evaluated in this study. When testing the kinship capabilities of these pipelines, relationships beyond the 5 th degree could not be assessed with the sampled family group. Both panels produced LRs exceeding 10^6^ for 2nd and 3rd degree relatives; however, only the Kintelligence Kit demonstrated medium-range kinship analysis capability for 4 th and 5 th degree relationships.

Kintelligence genotypes are compatible for upload to two law enforcement accessible genetic genealogy databases, GEDmatch PRO™ and FamilyTreeDNA, provided that more than 70% of SNPs are typed [70, 71]. These databases contain the SNP profiles of consenting individuals produced by direct-to-consumer genetic testing companies. In GEDmatch PRO™, UHR SNP profiles can be searched against the entire database, while SNP profiles derived from evidence in specific criminal cases and all cases uploaded to FamilyTreeDNA can only be searched against profiles that the consumer has opted in for law enforcement searching. [71, 72]

The GEDmatch PRO™ windowed kinship algorithm utilised for Kintelligence uploads has previously been shown to be an efficient and effective method for detecting and classifying relationships using simulated data and volunteer DNA samples [36, 73, 74]. This method produces a cM value, which estimates the total length of the genome shared between matched profiles, and a whole kinship coefficient to approximate relatedness. Furthermore, GEDmatch PRO™ facilitates database searching and direct comparisons to known profiles. If a known profile is generated from a reference sample using the Kintelligence Kit, it can be directly compared to unknown profiles using an LR generating tool, such as DBLR™ (used in this study) [45]. With the UAS v2.6 update, a kinship database can be constructed in-house using either the Kintelligence or Kintelligence HT kits. [75]

The FORCE panel has also been found to be suitable for extended kinship analysis in previous studies; however, the resulting profiles are not suitable for upload to law enforcement accessible genetic genealogy databases [15, 17, 56]. This workflow requires sequencing and analysing reference profiles for either direct comparisons or the building of an internal database to conduct direct or kinship searches. This pipeline could be particularly beneficial for localised identification efforts such as DVI, where references from family members of missing persons or antemortem samples can be collected and stored in a closed database [56]. Similar to Kintelligence, an LR generating tool can be used to compare profiles for extended kinship analysis.

## Conclusions

TAS technologies have been effectively implemented for unidentified and missing persons casework, presenting new investigative avenues in the absence of suitable antemortem or close familial reference samples. The SNP genotypes produced can be utilised for identification, inferring EVCs (i.e. hair, eye and skin colour), estimating BGA and extended kinship analysis. The Kintelligence Kit performed better for low DNA input samples than the FORCE panel with the QIAseq workflow in this study, with FORCE sequencing runs exhibiting a higher proportion of adapter dimers that were likely due to low amounts of template DNA for the majority of samples. There was substantial non-concordance between the Kintelligence and FORCE genotypes for the 992 shared SNPs, likely attributable to allele dropout in the FORCE panel. Additionally, the low DNA input for library preparation resulted in low coverage for FORCE sequencing runs and substantial locus dropout in the FORCE genotypes. Further optimisation of the FORCE panel with the QIAseq workflow may be required to ensure optimal performance for compromised samples where the DNA input is substantially lower than recommended amount.

Before validating an in-house forensic genomics pipeline, forensic laboratories must consider several key factors, including the facility and financial requirements, quality and quantity of DNA from typical forensic samples, availability of bioinformatics capabilities and genomics expertise and access to required databases for extended kinship analysis or the infrastructure to create their own. Additionally, laboratories should evaluate the types of genetic intelligence that can be derived from the genotype and whether it meets their operational needs. These two TAS pipelines offer in-house, end-to-end solutions for forensic genomics, ensuring transparency and accountability throughout the entire process.

## Data Availability

Data are stored at the Australian Federal Police and may be made available to approved entities upon written request and subject to consent provisions.
